# Experimental study of the catalytic effect of iron on low-rank coal gasification

**DOI:** 10.1038/s41598-022-09812-7

**Published:** 2022-04-06

**Authors:** Xuejun Qi, Shuang Lin

**Affiliations:** School of Architecture and Civil Engineering, Xihua University, Chengdu, 610039 China

**Keywords:** Coal, Chemical engineering

## Abstract

Acid-washing low-rank coal samples were loaded with different content of iron catalyst and then pyrolyzed. FT-IR, Raman spectra, and temperature-programmed experiments were used to investigate the influence of iron on the coal char. The FT-IR results revealed that iron catalyst rises the number of –OH, –CH_3_, and –CH_2_ functional groups. The Raman spectra results showed that partial large polyaromatic ring structures transform into small polyaromatic ring structures after the addition of iron. The results of temperature-programmed desorption indicated that the number of surface active sites is increased due to the addition of iron. For low-rank coal char with 3% Fe, the number of active sites increased with the increase of adsorption temperature until 800 °C and then start to decrease. At 750 °C, the adsorption capacity of CO_2_ increased with the increase of time and reached saturation state after 45 min. The results of the char-steam isothermal gasification experiment suggested that the iron catalyst enhances the gasification reactivity of low-rank coal. It is verified that iron catalysts can improve the gasification reactivity of low-rank coal by increasing the number of surface active sites.

## Introduction

Increased energy needs and usage cause increased emissions of harmful gas, such as CO_2_, NO_X_, and SO_X_. The release of these gases contributes to the greenhouse effect and the smog haze weather phenomenon. With the fast development of renewable energy, the consumption of coal is now decreasing. However, coal will continue to be an important component of the energy structure in China. The amount of low-rank coal is 2611.816 billion tons in China, accounting for about 57.38% of the country’s total predicted reserves^1^. Low-rank coal is characterized by high moisture content, high oxygen content, low heat, and flammability. The direct combustion of low-rank coal has low thermal efficiency and emits a large amount of dust, heavy metal pollution, and other pollutants, which has a greater impact on the environment than other coals. Hence, a clean utilization technology for low-rank coal is urgently needed to reduce the environmental problems of using this important resource. Coal gasification is an advanced technology to convert coal into fuel gas and synthesize gas for subsequent utilization^2,3^. The process of low-rank coal gasification with carbon dioxide or steam at high temperatures produced gaseous products of hydrogen and carbon oxides, a small amount of methane, and other light hydrocarbons. Gaseous products can be used in the system of Integrated Gasification Combined Cycle (IGCC), chemical synthesis, fuel cells, and fuel^4,5^.

Catalytic gasification in the coal conversion process is a promising way to develop significantly higher efficient coal conversion technology than non-catalytic gasification^6–8^. Using a catalyst in the process of coal gasification can decrease the required size of the reactor and enhance the reaction rate of coal and gasification agents. At the same time, tar and soot are removed during gasification, eliminating the problem of serious corrosion of gasification equipment caused by the presence of tar and soot^9^. Iron catalysts have a high catalytic effect for low-rank coal gasification^10–15^. Yu et al.^16,17^ studied the effect of a char-supported nano iron catalyst during coal-steam gasification and found that iron catalysts can be well dispersed in a char matrix and enhance the yield of hydrogen. Domazetis et al.^18^ investigated the catalytic steam gasification of brown coal containing iron species by both experiment and molecular simulation and found that Fe–C is the catalytic active site. Moreover, some researchers argued that FeCO_3_ can be used as a new type of iron catalyst during gasification, an economical option that reduces risk to gasification equipment and the environment^19,20^.

The release of volatiles and coal char gasification are the two main chemical reaction steps in the process of coal gasification, with the release rate of volatiles much higher than the coal char gasification rate. In the process of coal pyrolysis, numerous changes occur in the intraparticle pore structure, and there is a decrease in the active surface area of coal chars and a loss of functional groups on the carbon surface, greatly affecting the char gasification rate. The limiting step for coal gasification is the reactivity of coal char with the gasification agent^21^. The catalytic reactivity rate of coal mainly depends on catalyst types, reaction temperature, the distribution of catalysts, and the chemical forms of the catalysts^22,23^. Furthermore, gasification reactivity also has a close relationship with the structure of coal char, surface active sites, and specific surface area^24–26^. The previous study showed that iron catalysts can influence the carbon microcrystalline structure and gasification reactivity of Shenfu coal^27^. However, the influence of iron catalysts on the number of low-rank coal surface active sites remains unclear.

This paper aims to explore the effect of iron catalysts on the number of surface active sites of low-rank coal. Acid-washing low-rank coal samples were loaded with different content of iron catalysts before being subjected to a pyrolysis experiment under an N_2_ atmosphere. The change of functional groups and char structures were analyzed by FT-IR and Raman spectroscopy, respectively. The surface active sites were characterized by a temperature-programmed desorption (TPD) experiment. Finally, the gasification reactivity was investigated by a thermogravimetric analyzer.

## Experimental

### Low-rank coal samples and catalyst loading

The raw coal was partially dried at low temperature (< 323 K), then pulverized and sieved to obtain a fraction sample of particle sizes between 0.10 and 0.15 mm. The proximate and ultimate analyses for this coal are listed in Table 1. The raw coal ash composition analysis is shown in Table 2.

**Table 1:** Proximate and ultimate analyses of coal samples.

**Proximate analysis (wt% ad)**	
Moisture (M_ad_)	22.82
Ash (A_ad_)	10.43
Fixed (FC_ad_)	24.98
Volatile matter (V_ad_)	41.77
**Ultimate analysis (wt% daf)**	
Carbon	45.39
Hydrogen	4.93
Nitrogen	0.84
Sulfur	0.68
Oxygen	48.16

**Table 2:** Ash analysis of raw coal.

MgO	Al_2_O_3_	SiO_2_	SO_3_	Na_2_O
3.62	21.26	52.47	7.91	0.47
K_2_O	CaO	TiO_2_	MnO	Fe_2_O_3_
1.23	7.76	0.66	0.09	4.51

In order to eliminate the influence of other metals on char gasification reactivity, coal samples were subjected to acid-washing treatment using hydrochloric acid and hydrofluoric acid. Briefly, raw coal was washed with 1 mol/L HCl for 24 h and then rinsed with deionized water over filter paper. Consecutively, aqueous (40%) HF was added to HCl-washed coal and this slurry was stirred for 24 h and then washed by deionized water. After washing, the demineralized coal was dried in an oven at 378 K. Fe(NO_3_)_3_·9H_2_O was used as the iron catalyst precursor, and iron loading in the coal was varied as 1 wt%, 3 wt %, and 5 wt%. The catalyst loading was performed as follows. First, Fe(NO_3_)_3_·9H_2_O was placed in a beaker, then deionized water was added and stirred for 3 h to make it fully soluble in water. Second, the coal was subjected to acid-washing by immersion in an aqueous solution of Fe(NO_3_)_3_ with stirring for 6 h, ensuring a uniformly mixed pulverized coal and catalyst solution. Finally, the mixture was placed in an oven at 378 K and allowed to dry for several hours.

### Char preparation

The pyrolysis experiments with pulverized coal were performed in a fixed bed reactor. The flow rate of N_2_ was 1 L/min and was controlled by mass flow meters. About 4.0 g coal sample was put in an alumina crucible. Under N_2_, the fixed bed was heated. Once the temperature of the fixed bed reactor reached 850 °C, the coal samples were promptly placed into the central part of the reactor for about 15 min, and then cooled down to room temperature under N_2_ atmosphere.

### Char characterization

The surface-active functional groups were analyzed by VERTEX 70 microscopic Fourier transform infrared analyzer. The carbon crystallite structure of coal chars was analyzed by LabRAM HR800 laser confocal Raman spectrometer at 50 mW laser power, 532 nm wavelength, scanning for 2 min, with a range of wave number of 800 cm^-1^–1800 cm^-1^.

Temperature-programmed desorption experiments were performed using a Chemisorb 2720 chemical adsorption temperature-programmed device. The experiments were done as follows. First, 50 mg pulverized coal was placed on a U-type quartz tube reactor. High purity helium (35 mL/min) was used to remove the residual air in the piping system for 20 min before the experiment. Then a U-type quartz tube was heated from room temperature to the set temperature by 25 °C/min. When the temperature reached the goal temperature, the high purity helium was switched to CO_2_ (35 ml/min) required for the high temperature chemical adsorption reaction of CO_2_. After a period of reaction time, some C(O) complexes were formed on the surface of the coal char. Different pretreatments of char samples were obtained by using different adsorption temperatures and adsorption times. Finally, the U-type quartz tube was cooled to room temperature under the CO_2_ atmosphere after the chemical adsorption reaction. Next, at room temperature, the CO_2_ gas was switched to helium. To clear the adsorption reaction chamber and the residual CO_2_ gas in the piping system, helium purging was performed for 20 min. Then, the U-type quartz tube was heated to 950 °C at a rate of 10 °C/min, and the temperature-programmed desorption experiment was performed. The weight of the char sample and the release of CO were continuously recorded by a computer.

### Steam gasification of char in TGA

Steam gasification experiments were carried out on an STA 449 F3 thermogravimetric analyzer (TGA; NETZSCH Company). About 5 mg char sample was placed in a crucible and heated at 20 °C/min in each experiment. The reactor was heated to 850 °C in high purity N_2_ atmosphere (60 ml/min). Once at 850 °C, the N_2_ was then switched to steam (60 ml/min) for isothermal gasification. The weight of the sample was continuously recorded by a computer during char-steam gasification.

Here, the carbon conversion rate (X) was calculated by the following expression:

1 $$\text{X} = \frac{{\text{W}}_{0}{-{\text{W}}}_{\text{t}}}{{\text{W}}_{0}{\text{-W}}_{\text{f}}}$$

where W_0_ is the initial mass of the char sample, W_t_ is the instantaneous char sample mass at any time, and W_f_ is the mass of iron catalyst and ash.

## Results and discussion

### FT-IR analysis of coal char

**Figure 1:**
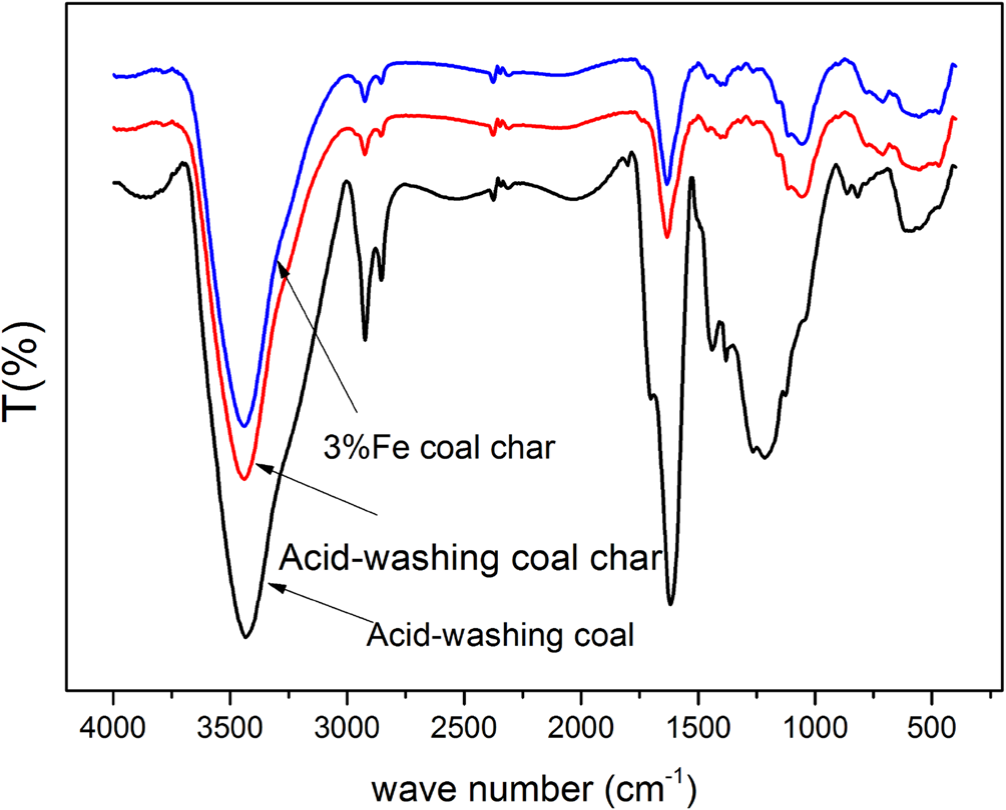
FT-IR spectrum of acid-washing coal and coal char.

The infrared spectra of acid-washing coal and coal char are shown in Fig. 1. From the data shown in the figure, it can be seen that strong absorption peaks appeared at 3430 cm^-1^(–OH), 2920 cm^-1^(–CH_3_ or –CH_2_), 2870 cm^-1^(–CH_3_ or –CH_2_), 1630 cm^-1^(C = C), and 1100 cm^-1^(C–O). Additionally, the absorption peak between 1330 and 1100 cm^-1^ represents C–O functional groups^28^. The strengthened absorption peak of OH, –CH_3_, and –CH_2_ indicate that coal char with iron catalysts produce more active functional groups than that of acid-washing coal char. This may be because the catalytic action of iron increases the cracking of the macromolecular structure of coal char into reactive functional groups. Reactive functional groups convert into condensed aromatic rings via polycondensation reactions after pyrolysis. Previous studies suggested that the Fe atom combines with functional groups of coal structure during pyrolysis to produce C–O–Fe or C–O–O–Fe chemical bonds, causing cracking of organic functional groups and forming other functional groups^29,30^.

### Raman analysis of coal char

**Figure 2:**
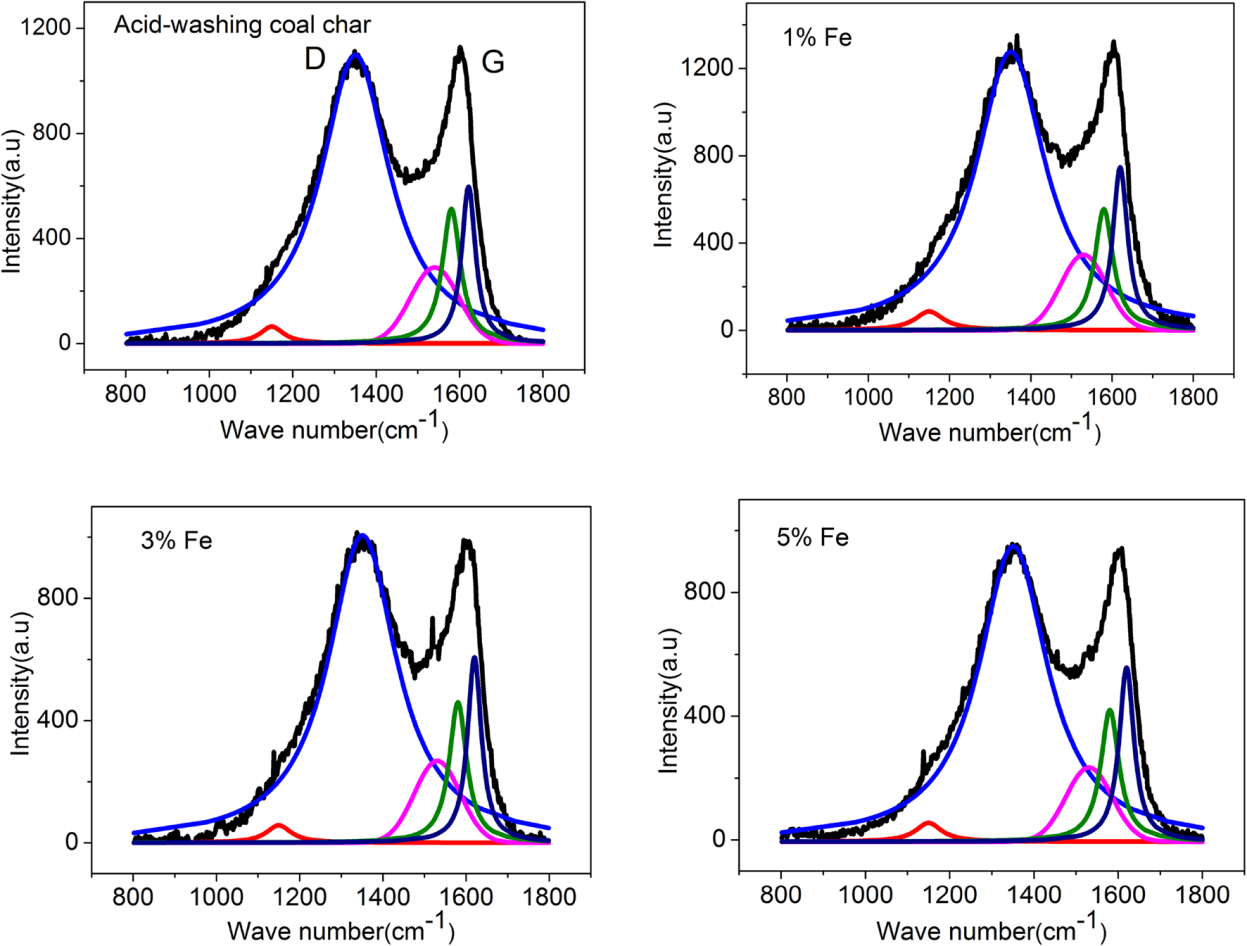
The fitting curve of the Raman spectrum.

The Raman spectra of coal char with different content of iron are shown in Fig. 2. The coal char shows two characteristic peaks (D peak and G peak). Due to the disordering of the low-rank coal char, detailed information about the carbon skeleton structure is hidden in the region around the D peak and the G peak. For this reason, the Raman spectra was divided into four Lorentzian bands and one Gaussian band using Origin8.6/Peak Fitting Module to get detailed information about the carbon skeleton structure of char. The D1 band (1350 cm^−1^) commonly represents the defects in graphite structure and other disordered structures^31,32^. The D2 band (1620 cm^-1^) often appears with the D1 band, which is attributed to a lattice vibration involving graphene layers, and its intensity decreases with the increasing degree of organization^33^. The D3 (1530 cm^-1^) band is attributed to amorphous carbon in the coal char structure, such as the SP^2^ amorphous carbon structure^31^. This consists of a small aromatic ring structure (3–5 condensation benzene) and has a close relationship with the reactive sites of coal char^34^. The D4 band (1150 cm^-1^) often appears in very poorly organized carbon materials, such as in soot and carbon black^35^. The G band (1580 cm^-1^) is related to large aromatic ring structures^36,37^.

**Figure 3:**
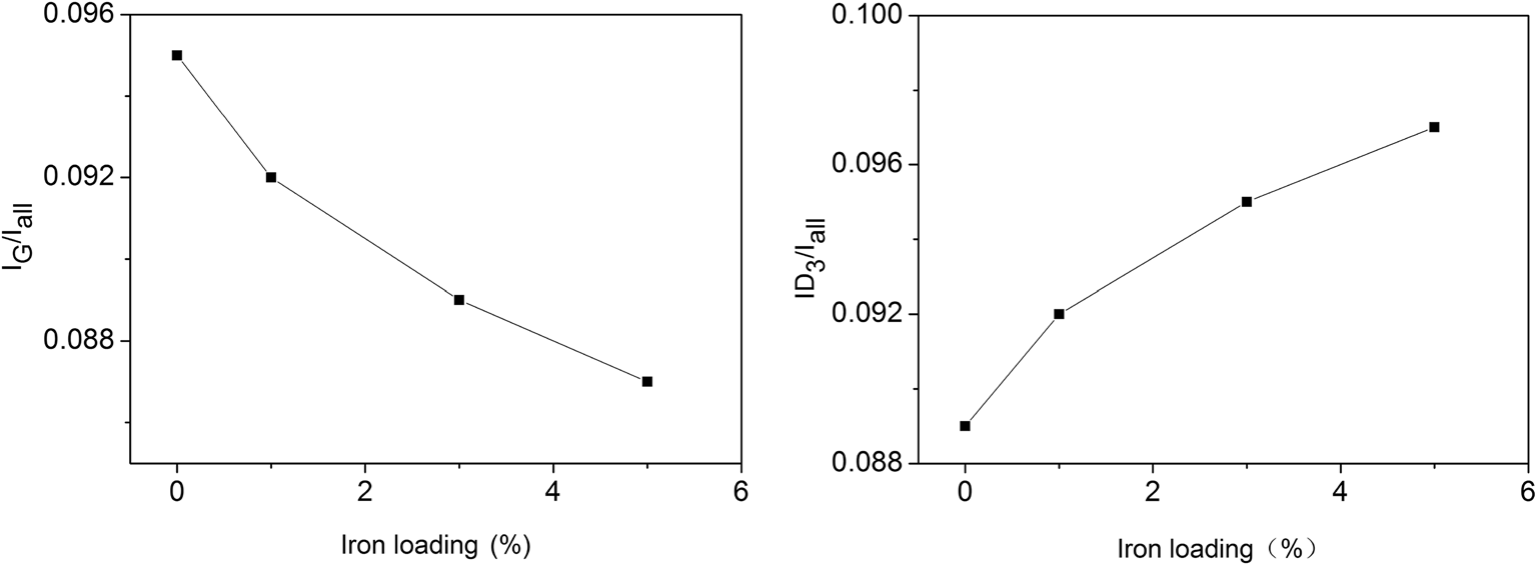
The variation trend of peak area ratio with the iron loadings.

The decomposition of Raman spectrum parameters allows the determination of the band intensity, full-width at half maximum, the peak position, and the integrated area of each peak. The ratio of the peak area is usually used to characterize the evolution of carbon crystalline structure. I_G_/I_all_ reflects the amount of polyaromatic structure and I_D3_/I_G_ indicates the content of amorphous carbon structure in coal char. To further apply the Raman parameters that correlate with the reactivities of coal char with the iron catalyst, the relationship between iron loading and the ratio of the G band to the integrated area (I_G_/I_all_) and the D3 band to the integrated area (I_D3_/I_all_) are illustrated in Fig. 3. It can be seen that I_G_/I_all_ decreases from 0.095 to 0.087 with the increase of iron loading. This means an increase in the number of the small aromatic ring structures and a decrease in the number of the large aromatic ring structures. It may be because Fe atoms insert into the structure of the char matrix and break the original C–C bonds to form small aromatic ring structures, which increase the disordering of coal char. At the same time, I_D3_/I_all_ increases from 0.090 to 0.097 with the increase of iron loading, which indicates produce more small molecule structures with 3–5 polycondensation benzene rings and amorphous carbon structures, which is related to the number of reacting active sites^38–40^. The decrease of I_G_/I_all_ and increase of I_D3_/I_all_ indicate that iron has a catalytic effect on the char structure during pyrolysis, which speeds up the cracking of coal char and improves the reactivity of coal char.

### The influence of iron on surface active sites of coal char

**Figure 4:**
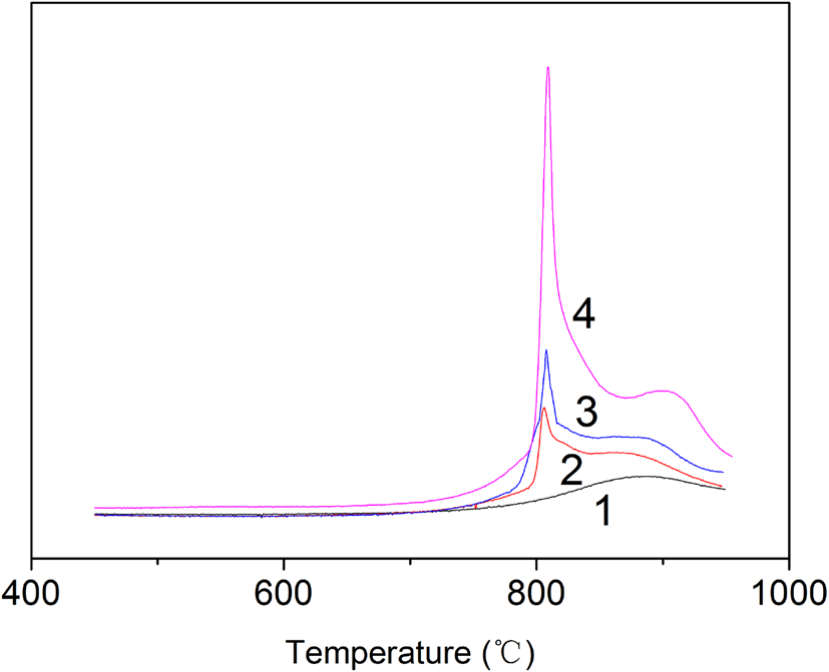
Effect of iron loading on release peak of CO. 1 Acid-washing coal char; 2 Coal char with 1% Fe; 3 Coal char with 3% Fe; 4 Coal char with 5% Fe.

The number of surface active sites on low-rank coal char is correlated with the gasification reactivity, which can be characterized by temperature-programmed desorption (TPD). According to the mechanism of the carbon–oxygen reaction, certain carbon atoms from the char structure can chemisorb an oxygen atom form a surface oxygen complex C(O) and release a gas phase CO molecule. The linear relationship between the release quantity of CO and surface active sites allowed the determination of the number of surface active sites as shown by the yield of CO. Figure 4 shows the influence of iron catalyst on surface active sites of coal char. The first weak release peak of CO appeared at 760 °C for acid-washing coal char. For coal char with an iron catalyst, the release peak appeared in the range of 795–820 °C. Moreover, the release quantity of CO continues to increase with the further increase in iron loading. This is likely because more iron atoms insert into the coal matrix and destroy the coal structure with the increase of iron loading. Hence, many active sites are formed on the char, which promotes the chemisorption of CO_2_ and the formation of surface C(O) complexes. Therefore, it can be concluded that the number of active sites is increased by the addition of an iron catalyst.

**Figure 5:**
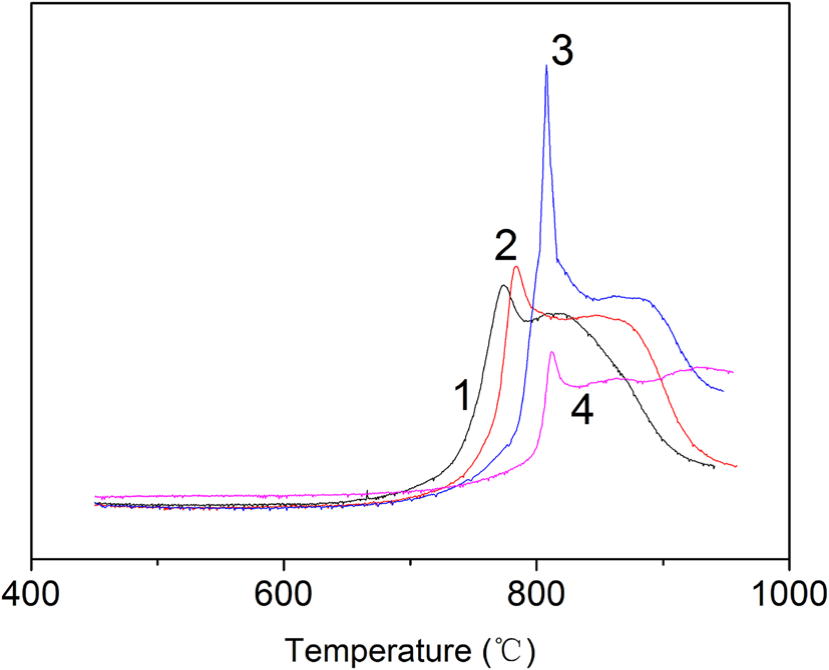
Effect of adsorption temperature on release peak of CO. 1–700 °C; 2–750 °C; 3–800 °C; 4–850 °C.

The sample of coal char with 3% Fe was used to study the influence of adsorption temperature on the number of active sites of coal char. High temperature chemisorption of CO_2_ on char was performed for 15 min for each experiment, and the results are shown in Fig. 5. It can be seen that the release peak of CO increases with the increase of adsorption temperature and began to decreases after 800 °C. This may be due to the gasification reaction between coal char and CO_2_ above 800 °C, which consumes the active sites and leads to the reduction of CO.

**Figure 6:**
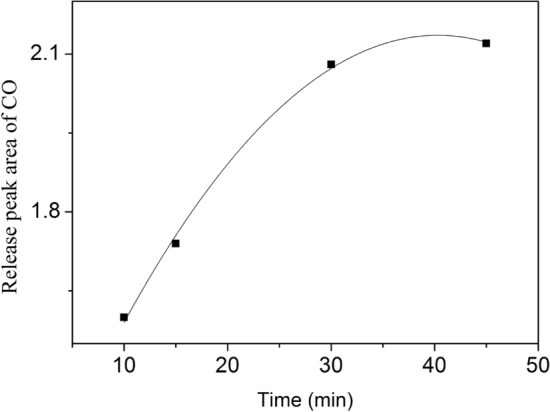
Effect of adsorption time on the release peak area of CO.

Next, coal char with 3% Fe was used to study the impact of adsorption time on the number of surface active sites. The high temperature chemisorption reaction was performed with different adsorption times (10, 15, 30, and 45 min) at 750 °C. Figure 6 presents the relationship between the release peak area of CO and adsorption time. The release peak area of CO represents the quantity of CO. From the figure, it can be seen that the quantity of CO shows a gradual increase with the increase of adsorption time, and the release curve of CO levels off after 45 min. This indicates that coal char reach the saturated adsorption state.

### Effect of iron catalyst on char-steam isothermal gasification

**Figure 7:**
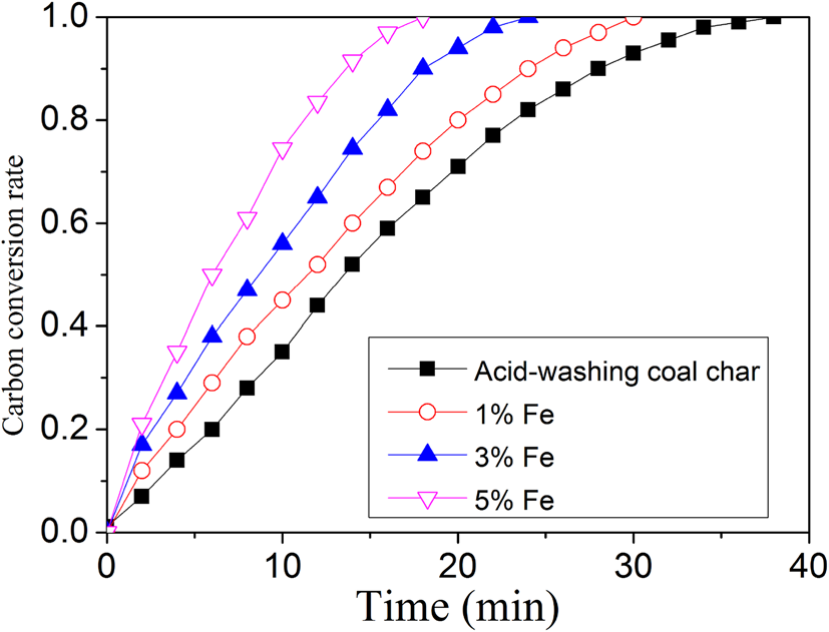
Effect of iron loading on coal char gasification reactivity.

Figure 7 illustrates the effect of iron loading on the carbon conversion rate during char-steam isothermal gasification at 850 °C. As shown in this figure, the carbon conversion rate of the catalyzed char was higher than that of the char without iron catalyst and the carbon conversion rate increased with the increase of iron loading. The low gasification reactivity of un-catalyzed coal char may be due to the removal of Na, K, Ca, and other catalytic minerals by the acid-washing treatment^41^. Many large condensed aromatic ring structures exist in coal char limiting the gasification rate. Unlike an un-catalytic reaction, in the gasification process of char with catalyst, iron atoms insert into the lattice structure to generate a C_x-1_–Fe–C_x_ structure. This structure belongs to a small aromatic ring structure and priority reacts with gasification agent. Hence, iron catalysts could accelerate the gasification rate by inhibiting the degree of graphitization^42,43^. It seems clear that the presence of iron catalyst exerts a catalytic effect on coal char and produces numerous small aromatic ring structures. According to the results of Raman analysis and TPD, char with iron catalyst contain many small aromatic ring structures, correlate with reactive active sites. The char-steam gasification experiment again verified the result of Raman analysis and TPD experiment. Taken together, these results suggest that iron catalyst exerts catalytic action on low-rank coal char is through increasing the number of surface active sites.

## Conclusions

1. In the process of pyrolysis, iron catalyst speeds up the decomposition of the macromolecular structure of coal char and produces more functional groups, such as OH, –CH_3_, and –CH_2_.
2. The presence of iron catalyst resulted in the conversion of large aromatic ring structures to small aromatic ring structures and increased the amount of amorphous carbon structure in coal char.
3. For coal char with 3%Fe, the number of active sites of coal increased with the increase of adsorption temperature and began to decrease after 800 °C. The quantity of CO showed a gradual increase with the increase of adsorption time, and the release curve of CO levels off after 45 min.
4. The gasification reactivity of coal char exhibited a close relationship with the char structure and surface active sites. The catalytic effect of iron is by increasing the number of active sites on the low-rank coal char surface.

## Data availability

The raw data required to reproduce these findings cannot be shared at this time as the data also forms part of an ongoing study.

## References

[1] http://www.gn580.com/readnews48995

[2] Irfan MF, Usman MR, Kusakabe K (2011). Coal gasification in CO_2_ atmosphere and its kinetics since 1948: a brief review. Energy.

[3] Yang Z, Hu J (2019). Catalytic steam gasification of Mengdong coal in the presence of iron ore for hydrogen-rich gas production. J. Energy Inst..

[4] Minchener AJ (2005). Coal gasification for advanced power generation. Fuel.

[5] Liu G-S, Niksa S (2004). Coal conversion submodels for design applications at elevated pressures. Part II. Char gasification. Prog. Energy Combust. Sci..

[6] Sharma A, Takanohashi T, Saito I (2008). Effect of catalyst addition on gasification reactivity of HyperCoal and coal with steam at 775–700°C. Fuel.

[7] Shen L, Nakamura A, Murakami K (2018). Effect of Fe_2_O_3_ on steam gasification of subbituminous coal/woody biomass mixture. Energy Sci. Eng..

[8] Jiang Y, Yan H, Guo Q (2019). Multiple synergistic effects exerted by coexisting sodium and iron on catalytic steam gasification of coal char. Fuel Process. Technol..

[9] He Q, Guo Q, Umeki K (2021). Soot formation during biomass gasification: a critical review. Renew. Sustain. Energy Rev..

[10] Ohtsuka Y, Asarni K (1997). Highly active catalysts from inexpensive raw materials for coal gasification. Catal. Today.

[11] Ohme H, Suzuki T (1996). Mechanisms of CO_2_ gasification of carbon catalyzed with group VIII metals. 1. Iron-catalyzed CO_2_ gasification. Energy Fuels.

[12] Ohtsuka Y, Asami K (1991). Steam gasification of low-rank coals with a chlorine-free iron catalyst from ferric chloride. Ind. Eng. Chem. Res..

[13] Ohtsuka Y, Tamai Y, Tomita A (1987). Iron-catalyzed gasification of brown coal at low temperatures. Energy Fuels.

[14] Asami K, Ohtsuka Y (1993). Highly active iron catalysts from ferric chloride for the steam gasification of brown coal. Ind. Eng. Chem. Res..

[15] Yamashitat H, Tomita A (1993). Local structures of metals dispersed on coal. 5. Effect of coal, catalyst precursor, and catalyst preparation method on the structure of iron species during heat treatment and steam gasification. Ind. Eng. Chem. Res..

[16] Yu J, Tian FJ, McKenzie LJ, Li CZ (2006). Char-supported nano iron catalyst for water-gas-shift reaction: hydrogen production from coal/biomass gasification. Proc. Saf. Environ. Protect..

[17] Yu J, Tian F-J, Chow MC, McKenzie LJ, Li C-Z (2006). Effect of iron on the gasification of Victorian brown coal with steam: enhancement of hydrogen production. Fuel.

[18] Domazetis G, James BD, Liesegang J (2012). Experimental studies and molecular modelling of catalytic steam gasification of brown coal containing iron species. Fuel.

[19] Popa T, Fan M, Argyle M (2013). H_2_ and CO_x_ generation from coal gasification catalyzed by a cost-effective iron catalyst. Appl. Catal. A Gen..

[20] Zhang F, Xu D, Wang Y, Argyle MD, Fan M (2015). CO_2_ gasification of powder river basin coal catalyzed by a cost-effective and environmentally friendly iron catalyst. Appl. Energy.

[21] Jamil K, Hayashi J-I, Li C-Z (2004). Pyrolysis of a Victorian brown coal and gasification of nascent char in CO_2_ atmosphere in a wire-mesh reactor. Fuel.

[22] Li C-Z (2007). Some recent advances in the understanding of the pyrolysis and gasification behaviour of Victorian brown coal. Fuel.

[23] Yamashita H, Yoshida S, Tomita A (1991). Local structures of metals dispersed on coal. 2. Ultrafine FeOOH as active iron species for steam gasification of brown coal. Energy Fuels.

[24] Chen SG, Yang RT (1997). Unified mechanism of alkali and alkaline earth catalyzed gasification reactions of carbon by CO_2_ and H_2_O. Energy Fuels.

[25] Molina A, MondragÓn F (1998). Reactivity of coal gasification with steam and CO_2_. Fuel.

[26] Yasyerli N, Doġű T, Doġű G, Ar İ (1996). Deactivation model for textural effects of kinetics of gas-solid noncatalytic reactions “char gasification with CO_2_”. Chem. Eng. Sci..

[27] Qi X, Guo X, Xue L, Zheng C (2014). Effect of iron on Shenfu coal char structure and its influence on gasification reactivity. J. Anal. Appl. Pyrol..

[28] Gong X, Zhancheng G, Zhi W (2009). Variation of char structure during anthracite pyrolysis catalyzed by Fe_2_O_3_ and its influence on char combustion reactivity. Energy Fuels.

[29] Lemaignen L, Zhuo Y, Reed GP, Dugwell DR, Kandiyoti R (2002). Factors governing reactivity in low temperature coal gasification. Part II. An attempt to correlate conversions with inorganic and mineral constituents. Fuel.

[30] Holstein WL, Boudart M (1983). Transition metal and metal oxide catalysed gasification of carbon by oxygen, water and carbon dioxide. Fuel.

[31] Ferrari AC, Robertson J (2000). Interpretation of Raman spectra of disordered and amorphous carbon. Phys. Rev. B.

[32] Wang B, Sun L, Su S, Xiang J, Hu S, Fei H (2012). Char structural evolution during pyrolysis and its influence on combustion reactivity in air and oxy-fuel conditions. Energy Fuels.

[33] Jawhari T, Roid A, Casado J (1995). Raman spectroscopic characterization of some commercially available carbon black materials. Carbon.

[34] Schwan J, Ulrich S, Batori V, Ehrhardt H, Silva S (1996). Raman spectroscopy on amorphous carbon films. J. Appl. Phys..

[35] Dippel B, Jander H, Heintzenberg J (1999). NIR FT Raman spectroscopic study of flame soot. Phys. Chem. Chem. Phys..

[36] Beyssac O, Goffé B, Petitet J-P (2003). On the characterization of disordered and heterogeneous carbonaceous materials by Raman spectroscopy. Spectrochim. Acta A.

[37] Sadezky A, Muckenhuber H, Grothe H (2005). Raman microspectroscopy of soot and related carbonaceous materials: spectral analysis and structural information. Carbon.

[38] Xiaojiang L, Jun-ichiro H, Chun-Zhu L (2006). FT-Raman spectroscopic study of the evolution of char structure during the pyrolysis of a Victorian brown coal. Fuel.

[39] Li X, Hayashi J-I, Li C-Z (2006). Volatilisation and catalytic effects of alkali and alkaline earth metallic species during the pyrolysis and gasification of Victorian brown coal. Part VII. Raman spectroscopic study on the changes in char structure during the catalytic gasification in air. Fuel.

[40] Li X, Li C-Z (2006). Volatilisation and catalytic effects of alkali and alkaline earth metallic species during the pyrolysis and gasification of Victorian brown coal. Part VIII. Catalysis and changes in char structure during gasification in steam. Fuel.

[41] Wang J, Morishita K, Takarada T (2001). High-temperature interactions between coal char and mixtures of calcium oxide, quartz, and kaolinite. Energy Fuels.

[42] He Q, Yu J, Song X (2020). Utilization of biomass ash for upgrading petroleum coke gasification: effect of soluble and insoluble components. Energy.

[43] Feng B, Bhatia SK, Barry JC (2002). Structural ordering of coal char during heat treatment and its impact on reactivity. Carbon.

